# Characterization and Inhibitory Effects of Magnetic Iron Oxide Nanoparticles Synthesized from Plant Extracts on HeLa Cells

**DOI:** 10.1155/2020/2630735

**Published:** 2020-12-10

**Authors:** Bernard Owusu Asimeng, Emmanuel Nyankson, Johnson Kwame Efavi, Amartey Nii Amarkai, Gloria Pokuaa Manu, Elvis Tiburu

**Affiliations:** ^1^University of Ghana, School of Engineering Sciences, Department of Biomedical Engineering, P.O. Box LG 74, Accra, Ghana; ^2^University of Ghana, School of Engineering Sciences, Department of Materials Science & Engineering, P.O. Box LG 74, Accra, Ghana

## Abstract

Magnetic Fe_3_O_4_ nanoparticles were synthesized from maize leaves and plantain peels extract mediators. Particles were characterized, and the inhibitory effects were studied on HeLa cells in vitro using cyclic voltammetry (CV). Voltammograms from the CV show that Fe_3_O_4_ NPs interaction with HeLa cells affected their electrochemical behavior. The nanoparticles formed with higher Fe^3+^/Fe^2+^ molar ratio (2.8 : 1) resulted in smaller crystallite sizes compared to those formed with lower Fe^3+^/Fe^2+^ molar ratio (1.4 : 1). The particles with the smallest crystallite size showed higher anodic peak currents, whereas the larger crystallite sizes resulted in lower anodic peak currents. The peak currents relate to cell inhibition and are confirmed by the half-maximum inhibitory concentration (IC_50_). The findings show that the particles have a different inhibitory mechanism on HeLa cells ion transfer and are promising to be further exploited for cancer treatment.

## 1. Introduction

Nanomedicine has shown a promising potential in clinical usage against a number of human diseases (cancer, diabetes, hypotension, etc.), with the development of nanoscale materials for use in biological applications, notably cancer therapeutics [1–5]. Among the numerous nanoscale materials for cancer diagnostics and treatment applications, magnetic nanoparticles (NPs) have emerged the most potent, and their magnetic properties have been exploited in magnetic resonance imaging (MRI), biosensors applications [6–12]. These magnetic nanoparticles in cancer treatment can be controlled to specific cancer sites for drugs to be delivered using external magnetics. Also, in the detection of cancer growth, MRI can be used to scan body parts or the materials can be designed by embedding them with receptors that are attracted to cancer cell.

Biological applications of magnetic nanoparticles have emerged as the material of interest due to their crystal inverse spinel crystal structure as well as their biocompatibility, superparamagnetic nature, and surface modification properties coupled with their unique physical, chemical, mechanical, and thermal features [13, 14].

Several methods have been employed in the synthesis of magnetic Fe_3_O_4_ nanoparticles (Fe_3_O_4_ NPs) including forced hydrolysis, hydrothermal technique, micro emulsion, and sol-gel methods. Co-precipitation synthesis approach is the simplest and most efficient method reported in literature [12, 15–18]. These chemical methods though have high yields of nanomaterials produced within a short time, they are expensive, and the chemicals involved are hazardous and produce toxic waste byproducts. There has been great interest in green synthesis of nanoparticles through biological means and its importance is well documented in literature [19, 20]. This interest is mostly fueled by the ability to synthesis nanoparticles without the use of toxic chemicals and involvement of hazardous byproducts.

Current novel approaches which are still under development use plant extract and other microorganisms such as algae, bacteria, actinomycetes, yeast, and fungi which results in environmentally friendly and biocompatible benign particles [18]. Plant extracts are generally much more of interest in biosynthesis because their throughput in nanoparticle production is much higher than the use of microbes and plant parts are also easily available [21, 22]. Plants naturally come equipped with bioactive molecules that act as reducing and stabilizing agents for inorganic iron salts employed in nanoparticle production [23, 24]. It is also reported that the nature of bioactive molecules present in plant extract has varying degrees of effect on the type and morphology of magnetic nanoparticles produced and they also influence the reactive mechanism of the metal salt reduction and stabilization process, especially where a combination of ferric (Fe^3+^) and ferrous (Fe^2+^) salts are used [25, 26]. The standard Fe^3+^/Fe^2+^ molar ratio for the formation of Fe_3_O_4_ nanoparticles to achieve the inverse spinel crystal structure is 2 : 1.

Maize and plantain are common tropical plants varieties whose fruits are consumed but their leaves and other parts are disposed as waste. In this work, we have synthesized Fe_3_O_4_ NPs from maize leaves and plantain peels using different molar ratios of Fe^3+^/Fe^2+^ and evaluated their biological interactions with HeLa cancer cells. The properties of the synthesized nanoparticles have been examined using x-ray diffraction (XRD), scanning electron microscopy (SEM), energy dispersive x-ray spectroscopy (EDX), UV-visible spectroscopy (UV-Vis), and Fourier-transform infra-red (FTIR). The biological interaction of these particles with HeLa cancer cells has been established using a cyclic voltammetry.

## 2. Materials and Methods

### 2.1. Raw Materials

All the reagents used in this work including ferrous chloride FeCl_2_.4H_2_O (M.W. 198.81), ferric chloride FeCl_3_.6H_2_O, and sodium hydroxide (NaOH) were of analytic grade and purchased from Sigma Aldrich, UK.

### 2.2. Preparation of Plant Extracts

Maize leaves, plantain leaves, and plantain peels used in this experiment were sampled from Ayido Flat E plantation farm on University of Ghana campus, Legon.

In preparing the extracts, each plant part was washed, cut into smaller sizes, and dried in a GeblabPrime oven at 60°C for 3 days, after which they were then ground/milled into powder using an Ika A111B mill. The grinding was done for approximately 10 minutes. 20 g of the powder was measured into a 300 mL beaker and 200 mL of distilled water added. The mixture was fluxed at 70°C for 1 hour, with continuous stirring. The mixture was then filtered with a cheesecloth and then with filter paper. The extract obtained was then stored at −4°C before use [27].

### 2.3. Synthesis of Magnetic M-Fe_3_O_4_ Nanoparticles

The preparation of Fe_3_O_4_ NPs followed an already established procedure in literature [28–30]. In this work, molar ratio FeCl_3_ hexa-hydrate and FeCl_2_ tetra-hydrate was used in a batch formation of 2.8 : 1 and 1.4 : 1 (Fe^3+^/Fe^2+^) mixture as shown in Table 1. The batch mixture of the precursors was dissolved in 200 ml of distilled water and stirred for 20 minutes. 40 ml of the extract was then added to the precursor solution changing the color of the solution from pale yellow to dark green (plantain peels extract) and light black (maize leaves extract). A 2.0 M NaOH solution at a rate of 3 mL/min was added for uniform formation of the magnetic Fe_3_O_4_ nanoparticles until pH of 11 was attained. The solution color changed to brown when pH was in the range of 3-4 and to black when pH was within 8–11. After complete nucleation of the nanoparticles, the black colored nanoparticles formed were labelled as plantain peels Fe_3_O_4_ NPs (PP-Fe_3_O_4_ NPs) and maize leaves Fe_3_O_4_ NPs (ML-Fe_3_O_4_ NPs). The nanoparticles were then immobilized using an external magnet. Distilled water was used to wash the particles 3 times and the washed particles were then freeze-dried.

### 2.4. Characterization of Fe_3_O_4_ Nanoparticles

The crystal growth planes and crystallite sizes were determined using x-ray diffraction (XRD). The analysis was conducted using X'Pert PRO x-ray Diffractometer (PanAnalytical) operating with Cu K-*α* radiation (wavelength of 1.544 Å) at 45 kV and 40 mA with a 20–70° 2*θ* range, a 0.2° step width scanning 1.2 deg/min on a 2 g sample. The absorbance was measured using a UV-Vis spectrophotometer (Thermo Fisher Scientific, China) operating within a scanning range of 300–800 nm with a 5 nm scan step on a 3 ml sample. The surface morphology of the samples was studied using a scanning electron microscope (SEM), Phenom desktop SEM with EDX analysis software. The Fourier-transform infra-red spectroscopy was performed using a PerkinElmer spectrum 2 spectrometer (PerkinElmer Inc., UK) with a 4000-400 cm^−1^ scanning range and 4 cm^−1^ resolutions on a 0.1 g sample.

### 2.5. Cell Viability Studies

HeLa cells were cultured in RPMI-640 medium supplemented with 10% FBS, 1% PS, and incubated at 37°C and 5% CO_2_. Cells (*n* = 3) were grown differently for 48 hours and each cell density was normalized using the inhibition rate factor given by (1)-(treated cell viability/untreated cell viability)^*∗*^ 100%. Thereafter, the nanoparticles were introduced into the cells and CV measurements were taken after 30 minutes. A quantity of 2.0 mg Fe_3_O_4_ nanoparticles (NPs) was mixed with 40 ml of phosphate buffer saline (PBS) and vortexed for 3 min to yield a stock solution. Volume of 5 *µ*L from the stock solution was added to 100 *µ*L of HeLa Cells. The mixture was allowed for 30 min incubation, after which 5 *µ*L was added to Ag/AgCl interdigitated electrode (DropSens, UK) for cyclic voltammetry (CV) analysis.

## 3. Results and Discussion

The results obtained from the characterization of the synthesized particle (ML-Fe_3_O_4_ NPs and PP-Fe_3_O_4_ NPs) and their effect on HeLa Cells (modelled cancer cells) are displayed and discussed. Fourier-transform infra-red spectroscopy (FTIR) x-ray diffraction (XRD), scanning electron microscopy (SEM), energy-dispersive x-ray spectroscopy (EDX), and cyclic voltammetry (CV) characterizations are reported below.

### 3.1. Fourier-Transform Infrared Spectroscopy (FTIR)

**Figure 1:**
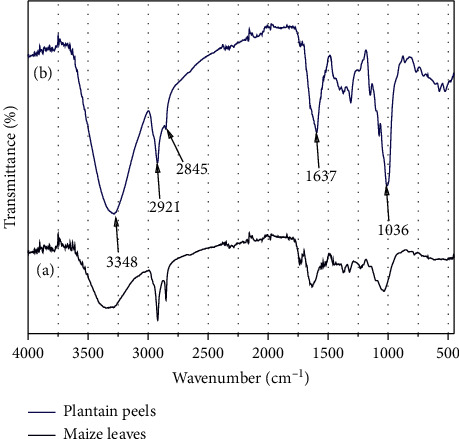
FTIR plot of extracts used in the synthesis. (a) Maize leaves. (b) Plantain peels.

FTIR spectroscopy studies were used to identify the functional groups associated with bioactive molecules present in the maize leaves and plantain peels extract which acted as both reducing and stabilization agents in the synthesis of the Fe_3_O_4_ NPs. The FTIR spectra of synthesized NPs obtained were within 3500-450 cm^−1^. The spectra of the extracts shown in Figure 1 revealed strong absorption bands at ∼3348 cm^−1^, ∼2921 cm^−1^, ∼2845 cm^−1^, ∼1637 cm^−1^, and ∼1036 cm^−1^. The region of ∼3348 cm^−1^ displayed bands corresponding to–OH (of phenol compound) stretching vibrations. The absorption peaks at ∼2921 cm^−1^ and 2845 cm^−1^ are associated with the sp3 C–H stretching vibrations of the –CH_2_ functional group [31]. The peak at ∼1637 cm^−1^ represents N-H bending of amide group and the peak at ∼1036 cm^−1^ corresponds to C-N stretching of aliphatic amines [32]. It is also observed in Figure 1 that the strength of absorption or intensity is stronger for the plantain peels than the maize leaves indicating that the phytochemicals identified are rich in the plantain peels compared to the maize leaves.

**Figure 2:**
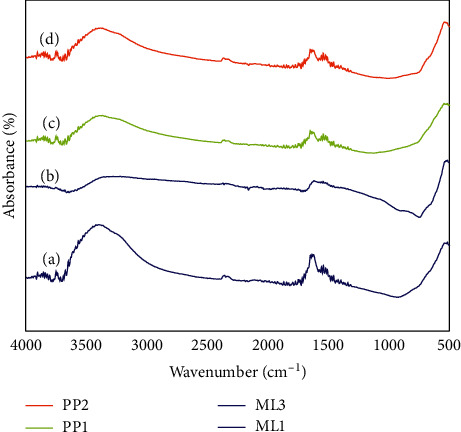
FTIR of plantain peels and maize leaves synthesized. (a) ML1-Fe_3_O_4_ NP. (b) ML3-Fe_3_O_4_ NP. (c) PP1-Fe_3_O_4_ NP. (d) PP3-Fe_3_O_4_ NP.

Absorbance bands of the synthesized Fe_3_O_4_ NPs were observed at ∼3340 cm^−1^, ∼1640 cm^−1^, ∼1063 cm^−1^, ∼900 cm^−1^, and ∼544 cm^−1^. The reduction in the intensity of band 3500 cm^−1^-3214 cm^−1^ in the extracts shows the involvement of the phenol compounds in the reduction of the ferrous and ferric chloride precursors. Based on the reduction in band intensities at ∼1637 and ∼1036 cm^−1^, proteins are also involved in the reduction of the ferrous and ferric chloride precursors. The presence of the peak at ∼544 cm^−1^ corresponds to stretching vibrations of Fe-O bonds depicting the presence of Fe_3_O_4_ [33]. The FTIR results, shown in Figures 1 and 2, all indicate that the bioactive elements present in the maize leaves and plantain peels leaves extracts could have been reducing and stabilizing agents in the synthesis process. The proteins and phenol compounds are believed to have been involved in the formation of Fe_3_O_4_. The formation and unique characteristics of Fe_3_O_4_ are as a result of electron transfer between Fe^3+^ and Fe^2+^ ions in tetrahedral and octahedral sites of the crystal lattice formed [28, 29]. It is established that a molar ratio of 2 : 1 (Fe^3+^/Fe^2+^) mixture is a precondition for the formation of Fe_3_O_4_ NPs due to reaction mechanisms [30, 34]; however, the crystallization process may be affected by the presence of phytochemicals in the plant extract acting as stabilization and reducing agents in the reaction mechanism which can lead to variation in the molar ratios required in the formation of Fe_3_O_4_ nanoparticles.

### 3.2. X-Ray Diffraction (XRD)

**Figure 3:**
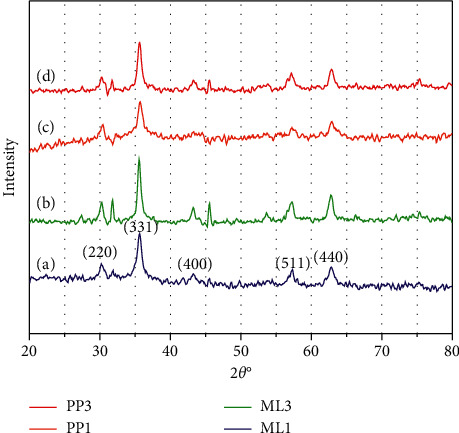
XRD pattern of Fe_3_O_4_ NPs synthesized with molar ratios 2.8 : 1 and 1.4 : 1. (a) ML1-Fe_3_O_4_ NPs. (b) ML3-Fe_3_O_4_ NPs. (c) PP1-Fe_3_O_4_ NPs. (d) PP3-Fe_3_O_4_ NPs.

Fe_3_O_4_ NPs were characterized by x-ray powder diffraction and patterns were collected to identify phases and crystalline structure and the results are presented in Figure 3. In total, it was found that there were intense diffraction peaks indexed (220), (311), (400), (422), (511), (440), and (533) at 2 theta values of 30.1°, 35.5°, 43.1°,54.5°, 57.6°, 62.8°, and 74.2°, respectively. The standard XRD data for magnetic iron oxide having a face-centered cubic structure was similar to the recorded data. The different molar ratios of 2.8 : 1 and 1.4 : 1 of Fe^3+^/Fe^2+^ used in the synthesis affected crystallites sizes of the nanoparticles obtained. It is established that the formation and unique characteristics of Fe_3_O_4_ are as a result of electron transfer between Fe^3+^ and Fe^2+^ ions in tetrahedral and octahedral sites of the crystal lattice formed [28, 29]. It is established that a molar ratio of 2 : 1 (Fe^3+^/Fe^2+^) mixture is a precondition for the formation of M-Fe_3_O_4_ NPs due to reaction mechanisms [30, 34]. It is also established that the crystallization process may be affected by the presence of the stabilization and reducing agents in the reaction mechanism leading to variation in the molar ratios required in the formation of Fe_3_O_4_ nanoparticles synthesized from green bioactive sources.

With the aid of the Debye–Scherrer relation, crystallite sizes were estimated using

(1) $$\begin{array}{c} D=\frac{0.89\lambda }{\beta \;\cos\;\theta }, \end{array}$$

where *D* represents the average crystallite size, *λ* represents the wavelength of the Cu-K irradiation, *β* represents FWHM of the most intense of the peak, and *θ* represents the diffraction angle of the (311) peak of the magnetic Fe_3_O_4_ NPs.

Table 2 presents the extracted peak intensity position and calculated crystal sizes. The crystallite sizes calculated from the XRD data indicate that for a decrease in the molar Fe^3+^/Fe^2+^ ratio the crystallite sizes increased.

It can be observed that the crystallite sizes of the Fe_3_O_4_ NPs formed with higher Fe^3+^/Fe^2+^ (2.8 : 1) molar ratios are smaller compared with those formed with lower ratios of Fe^3+^/Fe^2+^ (1.4 : 1). The crystallite size of Fe_3_O_4_ NPs has been prepared from *Cynara cardunculus* leaf using ferric chloride and ferrous chloride of a molar ratio of 1 : 2 M and obtained crystallite size of 13.5 nm [31]. The result is similar to the crystallite size obtained in this work for Fe_3_O_4_ NPs extracted with lower molar ratios of Fe^3+^/Fe^2+^ (1.4 : 1). An appreciable decrease in the crystallite size is observed when the molar ratio of the precursors is increased to a high malar ratio (2.8 : 1) and the observation is in line with works reported in literature [32, 33]. The high molar ratio prevents good agglomeration of the crystallites and thus decreases the size.

### 3.3. Energy Dispersive X-Ray (EDX) and Scanning Electron Microscopy (SEM)

**Figure 4:**
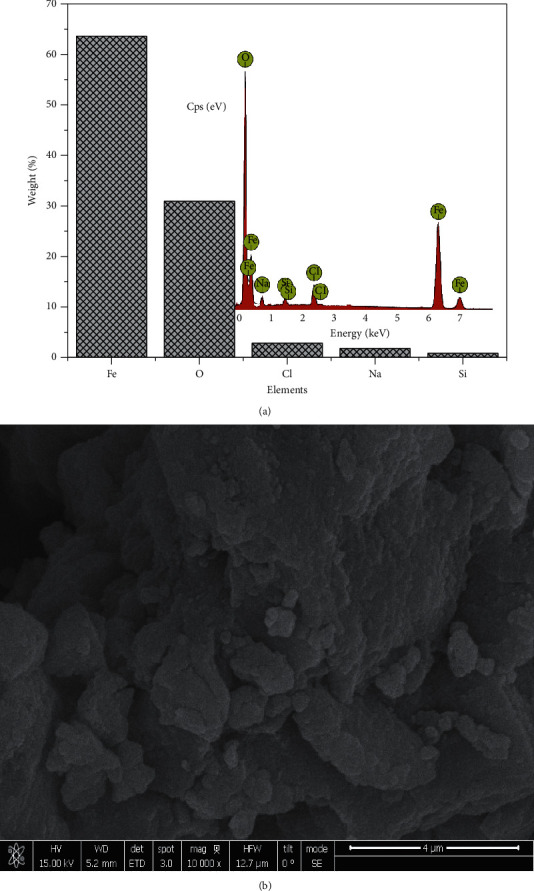
(a) EDX of ML1-Fe_3_O_4_ NPs. (b) SEM of ML1-Fe_3_O_4_ NP.

**Figure 5:**
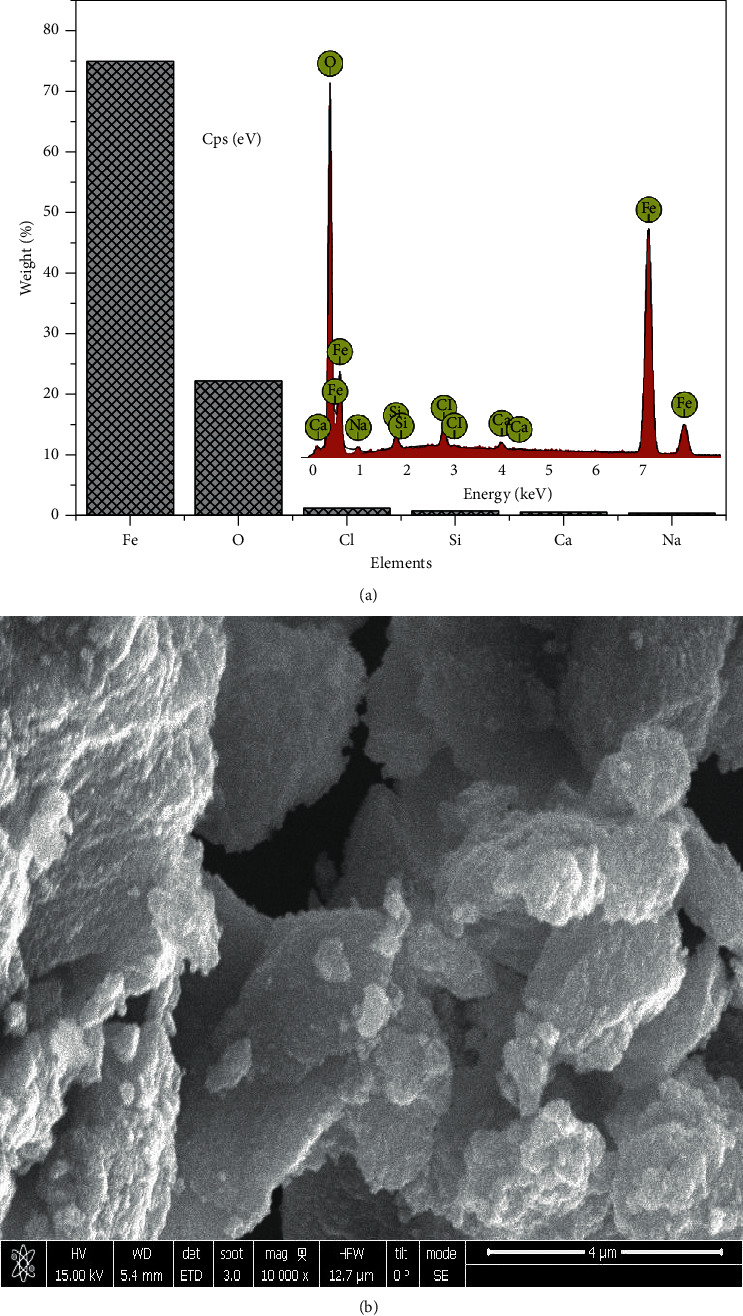
(a) EDX spectra of PP1-Fe_3_O_4_ NPs. (b) SEM of PP1-Fe_3_O_4_ NPs.

**Figure 6:**
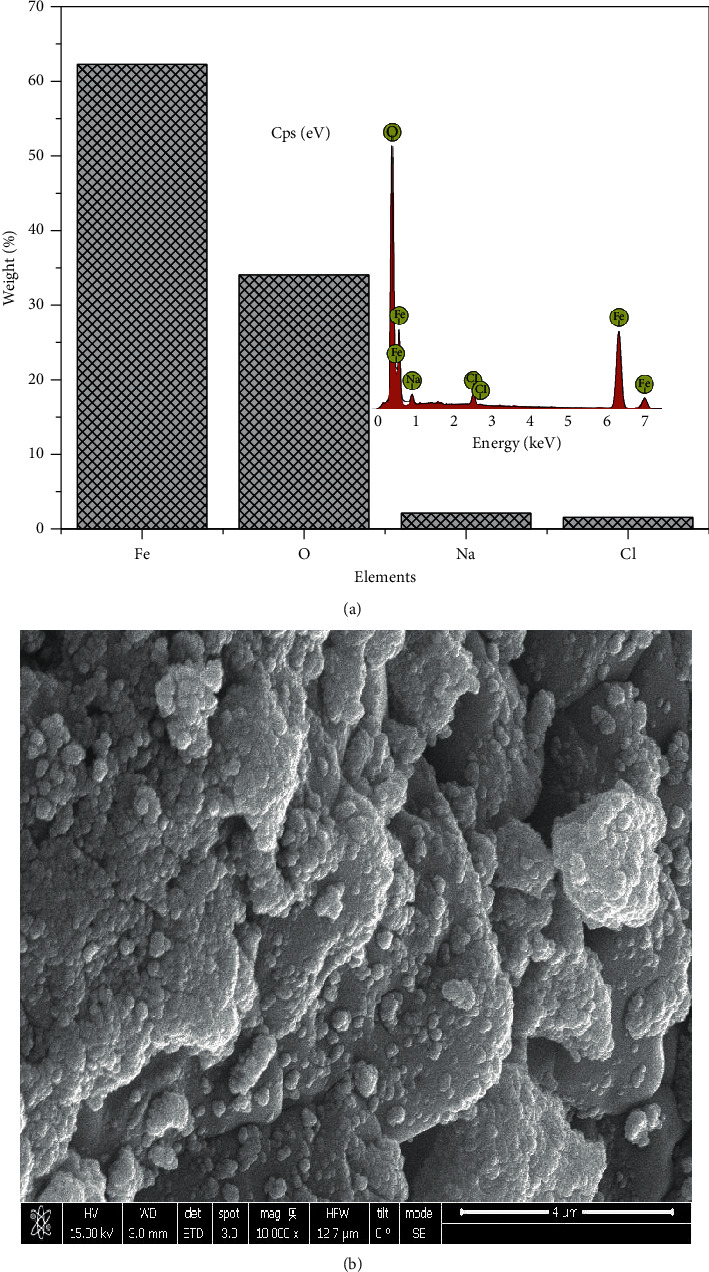
(a) EDX spectra of ML3-Fe_3_O_4_ NPs. (b) SEM of ML3-Fe_3_O_4_ NPs.

**Figure 7:**
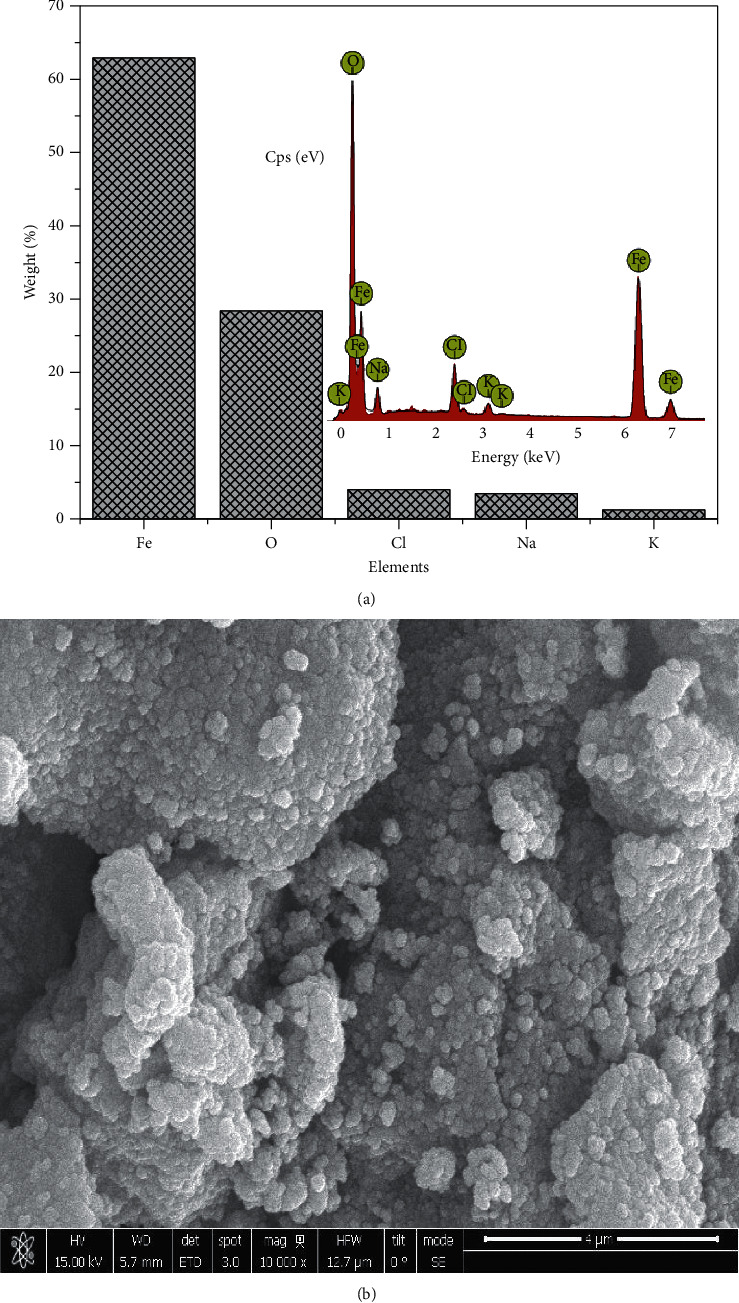
(a) EDX spectra of PP3-Fe_3_O_4_ NPs. (b) SEM of PP3-Fe_3_O_4_ NPs.

The EDX data obtained from the synthesized particles suggested in most of the particles formed that there were other elements besides Fe and O, mostly Na and Cl, which also was evident in XRD by the display of peaks around 2 theta angles of 32° and 45.5°. The presence of these peaks is as a result of Na in NaOH alkaline and Cl from the iron salts used in the synthesis. The EDX and SEM analysis of each sample are present in Figures 4–7. In addition, the EDX results in Figures 4(a), 5(a), 6(a), and 7(a) show the presence of other elements: calcium, silicon, magnesium, and potassium, which might be associated with the plant extracts.

The scanning electron microscopy (SEM) images display different texture and morphology for the different molar ratios of Fe^3+^/Fe^2+^ used in the Fe_3_O_4_ nanoparticles synthesized. The texture and morphology of ML1-Fe_3_O_4_ NPs and PP1-Fe_3_O_4_ NPs in Figures 4(b) and 5(b), respectively, are observed to be finer and agglomerated compared to a more coarser regular shaped ML3-Fe_3_O_4_ NPs and PP3-Fe_3_O_4_ NPs in Figures 6(b) and 7(b), respectively. These two distinct differences confirm the XRD crystallite size differences calculated and attributed to the differences in molar concentrations of the precursors and plant extracts used for the nanoparticle synthesis.

### 3.4. CV and IC_50_ Analysis of Cells with Fe_3_O_4_-NPs

**Figure 8:**
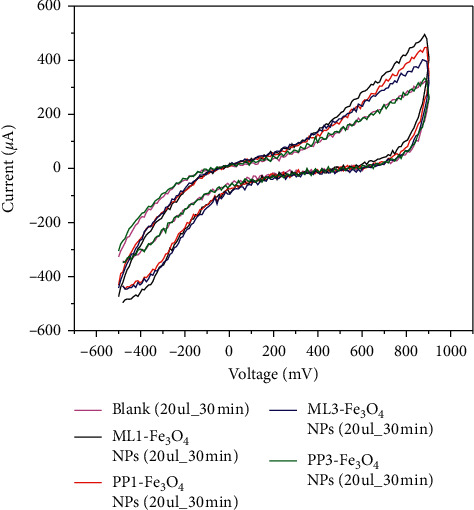
CV analysis plot after 20 *μ*l of sample suspension was added.

The cyclic voltammetry (CV) analysis conducted after every 30 min for 2 h displayed the influence of Fe_3_O_4_ NPs on Hela cells. The voltammogram (Figure 8) revealed variation of anodic peak current which corresponds to the different sources of Fe_3_O_4_ NPs suspension interaction with the blank (PBS).The voltammogram indicates that all the different sources of Fe_3_O_4_ NPs are REDOX active materials. There were available free sites in the Fe_3_O_4_ NPs to accept electrons from the electrode during the oxidation half cycle.

Interactions of Fe_3_O_4_ NPs suspension from different sources with HeLa cells also showed REDOX activities. ML1-Fe_3_O_4_ NPs showed higher anodic peak currents than the HeLa cells alone whereas ML3-Fe_3_O_4_ NPs showed peak currents lower than the HeLa cells alone. The peak current indicates that ML3-FeNPs proliferate through depolarization whereas ML1-Fe_3_O_4_ NPs proliferate through polarization. Cellular activities with cancerous cells are usual with depolarization [32]. Thus, polarization signal obtained suggests inhibition of cancerous proliferation. For PP1-Fe_3_O_4_ NPs and PP3-Fe_3_O_4_ NPs, the anodic peak current was similar to ML1-Fe_3_O_4_ NPs and ML3-Fe_3_O_4_ NPs, respectively. The influence of nanoparticles on HeLa cells correlated with concentration and nanoparticulate (crystallite size) size of particles. Variation of concentration returned different peak currents that gave different electrochemical gradients which resulted in wavy patterns. The patterns are similar to patterns of normal cellular activities [35]. The polarization of the cells corresponded to particles with smaller crystallite size whereas depolarization related to particles with larger crystallite sizes. It is suspected that the particles with the smaller crystallite size created high electrochemical gradient in the extracellular matrix for more ion outflow from the cytoplasm thereby inhibiting the cell depolarization signal. On the other hand, the larger crystallite particles are suspected to block the voltage gates of the ion channels. The blockage prevented more outflow of ions into the extracellular matrix.

**Figure 9:**
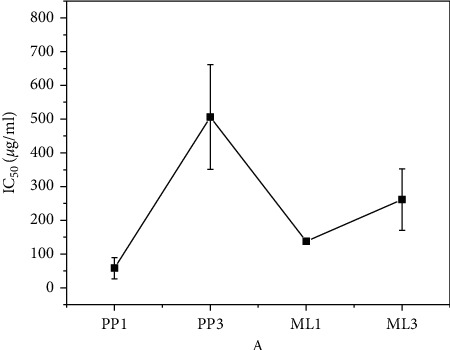
The particle potency to inhibit half of the HeLa cells. Low IC_50_ indicates high inhibitory potent.

From Figure 9, all the nanoparticles show inhibitory effect on the proliferation signal but with varying degrees; however, smaller calculated crystallites ML1-Fe_3_O_4_ NPs and PP1-Fe_3_O_4_ NPs exhibited much lower IC_50_ values. The differences in bioactive molecules in the extract are suspected to have played a role in the inhibitory potential. From the IC_50_ studies, PP1-Fe_3_O_4_ NPs is the most highly potent on the cells. The result showed that the particles with the highest potency are associated with smaller particle size.

## 4. Conclusion

The feasibility of synthesizing magnetic iron oxide particles from maize leave and plantain peels has been established. The precursor's molar ratio was varied to obtain four different particles: two from the maize leave (ML1-Fe_3_O_4_ NPs and ML3-Fe_3_O_4_ NPs) and the other two from plantain peels (PP1-Fe_3_O_4_ NPs and PP3-Fe_3_O_4_ NPs). The ML1-and PP1-Fe_3_O_4_ NPs were prepared from higher molar ratios whereas ML3-and PP3-Fe_3_O_4_ NPs were of a lower molar ratio. Crystallographic information determined from the XRD patterns showed that the ML1 and PP1-Fe_3_O_4_ NPs had lower crystallite size than that of ML3- and PP3-Fe_3_O_4_ NPs. These results affected the interaction of the nanoparticles with HeLa cells differently. The particles with the smallest crystallite sizes polarized the cells whereas the ones with the largest crystallite size depolarized the cells. IC50 studies show high potency of inhibition with the low crystalline particles compared to the large particles and, thus, the particles show potential in targeted cancer treatment.

## Figures and Tables

**Table 1:** The molar ratio of precursors used for the synthesis of nanoparticles.

Extract used	Fe^3+^ mass used	Fe^2+^ mass used	Fe^3+^/Fe^2+^	Sample name
Maize leaves	5.52	1.98	2.8 : 1	ML1-Fe_3_O_4_ NPs
	10.82	7.97	1.4 : 1	ML3-Fe_3_O_4_ NPs

Plantain peels	5.52	1.98	2.8 : 1	PP1-Fe_3_O_4_ NPs
	10.82	7.97	1.4 : 1	PP3-Fe_3_O_4_ NPs

**Table 2:** Magnetic nanoparticles prepared and their corresponding crystallite sizes.

Sample	FWHM	Peak position	Crystallite size (nm)
ML1-FeNPs	1.23489	35.59	7.05
ML3-FeNPs	0.62413	35.56	13.96
PP1-FeNPs	1.08648	35.72	8.02
PP3-FeNPs	0.71084	35.63	12.26

FWHM: full width half maximum.

## Data Availability

The data used to support this research's findings are included within the manuscript.
